# Psychomotor seizures, Penfield, Gibbs, Bailey and the development of anterior temporal lobectomy: A historical vignette

**DOI:** 10.4103/0972-2327.64630

**Published:** 2010

**Authors:** Prasad Vannemreddy, James L. Stone, Siddharth Vannemreddy, Konstantin V. Slavin

**Affiliations:** Department of Neurosurgery, University of Illinois at Chicago, Chicago, IL, USA

**Keywords:** Electroencephalography, epilepsy, ictus, temporal lobe, temporal lobectomy

## Abstract

Psychomotor seizures, referred to as limbic or partial complex seizures, have had an interesting evolution in diagnosis and treatment. Hughlings Jackson was the first to clearly relate the clinical syndrome and likely etiology to lesions in the uncinate region of the medial temporal lobe. With the application of electroencephalography (EEG) to the study of human epilepsy as early as 1934 by Gibbs, Lennox, and Davis in Boston, electrical recordings have significantly advanced the study of epilepsy. In 1937, Gibbs and Lennox proposed the term "psychomotor epilepsy" to describe a characteristic EEG pattern of seizures accompanied by mental, emotional, motor, and autonomic phenomena. Concurrently, typical psychomotor auras and dreamy states were produced by electrical stimulation of medial temporal structures during epilepsy surgery by Penfield in Montreal. In 1937, Jasper joined Penfield, EEG was introduced and negative surgical explorations became less frequent. Nevertheless, Penfield preferred to operate only on space occupying lesions. A milestone in psychomotor seizure diagnosis was in the year 1946 when Gibbs, at the Illinois Neuropsychiatric Institute, Chicago, reported that the patient falling asleep during EEG was a major activator of the psychomotor discharges and electrographic ictal episodes becoming more prominently recorded. Working with Percival Bailey, Gibbs was proactive in applying EEG to define surgical excision of the focus in patients with intractable psychomotor seizures. By early 1950s, the Montreal group began to clearly delineate causative medial temporal lesions such as hippocampal sclerosis and tumors in the production of psychomotor seizures.

## Introduction

The late 19^th^- and early 20^th^-century neurological and psychiatric literature detailed cognitive and behavioral pathologies in patients with epilepsy and diverse symptomatic presentations and etiologic theories.

Jackson[1] proposed the concept of "dreamy states" and related these symptoms to the lesions in the mesial temporal lobe structures and coined the word "uncinate fits." Thus, credit goes to Jackson for linking these epileptiform manifestations to the temporal lobe.

Although in the late 1930s, Gibbs and associates were likely the first to clearly describe the electroencephalographic (EEG) discharge patterns found in psychomotor seizures, they were initially led to faulty frontal or parasagittal localization of the focus by the infrequent use of an active temporal electrode and routine usage of a ear lobe reference electrode.[2,3] By 1946, working at the University of Illinois at Chicago (UIC), Gibbs recognized that the often temporally localized epileptiform discharges of psychomotor epilepsy (PME) were activated by a sleep EEG study. This resulted in an increased frequency of an electrographic diagnosis in these patients and led directly to the idea of a surgical approach to intractable cases. In 1937, Penfield brought Herbert Jasper and EEG to Montreal. Although initially skeptical about this new technology, Penfield's opinion changed when Jasper applied EEG to patients at the Montreal Neurological Institute (MNI).

Gibbs *et al*, observed a higher rate of psychiatric disorders among patients with psychomotor seizures than patients with other seizure types and in 1937 proposed the term "psychomotor epilepsy" to describe a characteristic EEG pattern of temporal lobe seizures, accompanied by mental, emotional, motor, and autonomic phenomena.[4]

EEG, first developed and reported by Hans Berger in 1929,[5] was accepted by the international community only after Adrain and Matthews, 1934, in England, confirmed Berger's original results.[6] Richard Caton, a British physician and physiologist, published his findings on the electrical phenomena of the exposed cerebral hemispheres of rabbits and monkeys in 1875 and they were rediscovered by Adolf Beck (1891) in Poland and later by Pavel Kaufman (1912) in St Petersburg.[7] Jasper, a doctorate in psychology, took interest in studying neurophysiology and published the first report in the United States in 1935, on research application of EEG[8] and in the same year the Boston group led by Davis, Lennox, and Gibbs had noted its clinical value in epilepsy.[9] The surgical treatment of epilepsy attracted more attention between 1945 and 1955.

## Jackson, Horsley, and the Beginning

During the Linacre Lecture given in 1909 on "The Function of the So-called Motor Area of the Brain"[10] at Cambridge University, Victor Horsley cited his report of 1886 on the first three cases of focal Jacksonian epilepsy he had operated upon during that time, primarily because he was urged and supported by Hughlings Jackson and David Ferrier. Jackson considered focal epilepsy as a manifestation of cortical irritation leading to a discharge of electrical energy. Thus, he suggested treatment by removal of the irritating focus[11] and prevailed on Horsley to perform craniotomy for the same.[12]

A direct support for Jackson's theory was provided when Horsley removed a highly vascular 3·2 cm scar along with a border of cortex. This showed the presence of a discrete cortical vascular scar and the focal motor seizures stopped postoperatively. Jackson stated, "there was in every case of epileptiform seizures a persisting discharging lesion." He believed that the starting point of the fit was a sign of the seat of the "discharging lesion," and advocated excision even if the region appeared normal.

Exhilarated by these beginnings of epilepsy surgery, Horsley made frequent reports of his experiences in brain surgery over the next decade.[10,13,14]

Between 1893 and 1912, another pioneer brain surgeon, Fedor Krause, operated on 96 patients for the treatment of epilepsy.[15] He stated that posttraumatic scars could produce "adhesions with the arachnoid, pia, and the surface of the brain" and these changes "could affect the cortical blood vessels present within bands of cicatricial tissue." Additionally, Krause also recognized that the primary spasmic center, the epileptogenic area, may be distant from the structural lesion and he considered the operative strategy in such cases, addressing "the question whether the morbid focus only should be excised, or in conjunction with it the primary spasming field as well." He added "in few instances I have adopted the latter plan with excellent result."

During World War I (WW I), many soldiers with posttraumatic epilepsy went to Otfrid Foerster in Breslau to seek relief. Like Krause, Foerster initially had no diagnostic or technologic means to localize the seizure and depended almost entirely on the chance observation of a seizure to exhibit a focal onset. In 1924, Foerster reported that hyperventilation precipitates an epileptic attack. It was also during this post WW I period that Berger, the German neuropsychiatrist, first recorded the EEG in veterans with cranial defects. He later found that such recordings could be done on intact skull and scalp and published these findings in 1929.[16]

## Wilder Penfield and Temporal Lobectomy

Foerster had observed that epileptic seizures can be initiated by electric stimulation around the meningocerebral cicatrix in post injury epileptics, or gentle traction on the cicatrix itself. He and Penfield (a visiting fellow) proposed that the resultant traction on the encased blood vessels might set up a "vaso-motor reflex secondary to this traction," which could be "responsible for the initiation of the convulsive seizures." Penfield became familiar with Foerster's use of local anesthesia and electrical stimulation to map motor and sensory areas of the human cortex exposed at operation in order to protect them during the excision of epileptogenic gliotic tissue.[17]

In November 1928, Penfield and his surgical partner, William Cone, performed their first operation in North America, utilizing the Foerster method. The patient, a young man previously treated for a head injury, had as many as 20 seizures each day and improved after three operations.[18]

## Frederic Gibbs and the Temporal Lobe

In 1936, Frederic Gibbs and William Lennox of the Boston school of EEG, classified psychomotor seizures as a separate entity from petit mal and grand mal for the first time. They considered epilepsy to be a paroxysmal "disordered functioning of the rate-regulating mechanism of the brain."[19] Accordingly, the presence of a structural lesion was more often an exception than a rule, and the various types of seizures would be considered as variations of the pattern of dysrhythmia.[2]

The Gibbs-Gibbs-Lennox team of the late 1930s generated a body of work of unprecedented achievement in clinical epileptology. Twelve children with petit mal epilepsy were the clinical subjects for the groundbreaking studies which began in late 1934 recognizing the association of petit mal absences with 3/s spike-wave complexes.[9,19,20]

Following extensive animal work, they investigated the relationship of EEG, cerebral blood flow, and blood constituents including carbon dioxide, oxygen and glucose in humans.[21] In 1938, Gibbs and Lennox described the first ever case on record in which operation was advised and a portion of brain removed based solely on the EEG findings of a focal epileptiform discharge.[22] From their experiences obtained by 1939, the sobering fact was evident that a sharply localized epileptic focus can be removed and the epilepsy still remains. Thus, Gibbs and Lennox assumed that in some cases, the cortical focus was not the only disorderly region!

From 1940s onward, the close integration between neurophysiology and neurosurgery, a mirror of the friendship between Penfield and Jasper, made the MNI a leading world center for epilepsy surgery. Other centers, such as the epilepsy surgery program at the UIC, began in 1944, when Frederic Gibbs arrived from Boston to work with Percival Bailey (1892-1973) and others.[22] By 1946 Gibbs *et al*, confirmed that cerebral blood flow in humans was not decreased in epilepsy, as popularly believed, but rather increased during the seizure.[23] This was in accordance with cerebral engorgement and "reddened veins" observed at surgery during seizures by Penfield and others.

## Penfield, Jasper, Gibbs, and Bailey

Although by 1941 Jasper was aware that mesial temporal structures might play a role in the origin of PME, one of the major obstacles for their excision was the lack of knowledge about their function. Jasper and Kershman wrote:

It seems clear from the nature of these disturbances that the temporal lobe and subjacent structures, probably in the archipallium, are the regions primarily involved. This is in accord with the electrographic localization, which so frequently seems to be deep to the temporal lobes (e.g., the hippocampus) near the midline or to involve an area first in one temporal lobe and then in the other.[24]

Such secondary epileptiform foci, known today as "mirror image foci," are believed to be "kindled" by very active primary focal discharges and are not uncommonly detected and studied with surface or depth EEG epilepsy protocols.

Focal pathology, such as uncommon calcified hemangiomas in the temporal lobe, might show paroxysmal discharges on scalp EEG and abnormal waves localized to the area of the temporal lesion at operation. But if a visible lesion was not found at the place indicated by the recording of epileptic activity, Penfield declined to perform resection. Because of this approach, about 20% of "exploratory craniotomies" for epilepsy were closed without cortical removal. The success rate of just over 50% in the MNI surgical series indicated that resection limited to the anterolateral temporal cortex did not eliminate all the epileptogenic tissue in many patients.[25] Penfield and Flanigin reviewed 68 patients (1939-1949) who had surgery for temporal lobe seizures at the MNI with 1 year of follow-up. In this series, 10 patients had the uncus removed and 2 also had hippocampal removal. The seizures were controlled in half of them. Electrophysiological abnormalities, such as spike activity and slow waves, were often recorded from the temporal cortex in this series,[26] but an anatomic substrate was not properly substantiated. Persistent seizures in some cases led Penfield to carry out a second operation to extend the mesial resection of the temporal lobe to eliminate the spike activity shown on the ECoG. The suction removal of these structures also made them unavailable for pathologic examination. Murray Falconer provided a solution by *en-bloc* excisions.[27]

Meanwhile, at the UIC, Gibbs *et al*,[4] assigned the origin of psychomotor seizures to the anterior and lateral cortex of the temporal lobe based on EEG studies.

Bailey and Gibbs reported their first 25 operations (1947-1950) guided by EEG and ECoG; these resections were confined to gyrectomies in the lateral and anterior aspects of the temporal lobe. Like Penfield, Bailey was an expert neuropathologist and looked for visible lesions at the epileptogenic sites localized by ECoG.[28]

In their initial series, Bailey reported opaqueness and thickening of the leptomeninges, atrophy of the convolutions, and scarring of old contusions in the removed tissue. In no instance did his extirpation extend to the insula or to the upper bank of the lateral fissure; he also carefully avoided the hippocampus.

Bailey was concerned to avoid the severe deficits demonstrated after mesial bitemporal resections in monkeys, a phenomenal observation made at the University of Chicago by several of Bailey's associates in the late 1930s.

Heinrich Kluver and Paul Bucy[29] published results of their experiments on monkeys in which they resected both the temporal lobes. The resultant syndrome, named after the authors, was characterized by psychic blindness, compulsion, loss of emotional reaction, and increase in sexual activity, a syndrome that would be disastrous if produced by surgical resection in patients. The findings in monkeys led Bailey to restrict his excisions to the anterolateral cortex of the temporal lobe.[30] Interestingly, although Bailey and Gibbs had a multicontact depth electrode designed by Erna Gibbs and Robert Hayne, which was occasionally employed intraoperatively, there is no evidence that they implicated the mesial temporal lobe structures as foci of epileptiform discharges. They did not, in other words, modify the extent of temporal lobe resections performed (Personal communication: Oscar Sugar)

## The Medial/Mesial Temporal Lobectomy

In his analysis, Brodal[31] excluded the hippocampus from the olfactory system and Kaada[32] produced attacks of arrest of movement, licking, chewing, and swallowing by stimulating the amygdala, the head of hippocampus, and the pyriform region in animals. To understand seizure propagation, other studies investigated the connections of mesial temporal structures with the rest of the brain.[33]

Convincing physiologic evidence that the mesial temporal region was a crucial zone for the generation of temporal lobe seizures came from the systematic reproduction in 16 patients of habitual auras and other typical features of their attacks, by depth stimulation at operation within and around the amygdala, involving the ventral claustrum and the anterior insula also sometimes.

The responses were emotional, visceral, impaired consciousness and confused memory, or brief seizures with automatism and amnesia.

Depth stimulation of the amygdala in cats reproduced these electrographic features of widespread cortical seizure activity, similar to responses produced by stimulation of the brainstem reticular formation. These findings demonstrated that the epileptic activity recorded from the lateral and anterior temporal cortex might be secondary to an epileptic focus deep in the claustroamygdaloid complex.[34]

The group from Montreal also proposed that "incisural sclerosis," the gross scarring and atrophy of the mesial temporal structures encountered by the surgeon, could result from an ischemic injury caused by tentorial herniation, with compression of the uncinate region and hippocampus, or compromise of their blood supply, associated with either difficult birth or childhood brain injury.[35] They based their theory on focal pathologic abnormalities found in a series of 157 patients with temporal lobe epilepsy operated upon by Penfield.

Penfield and Baldwin published their classical report describing the technique for subtotal temporal lobectomy including amygdala and hippocampal complex and concluded that "the abnormal, sclerotic area of the cortex, which must be removed in most cases, lies in the deepest, most inferior and mesial portion of the [temporal] lobe." Their report laid out the resection that would serve as the model for temporal lobe surgery.[30,31]

From 1953 onward, other neurosurgical centers took up anteromesial temporal lobe resection for the treatment of PME.[36,37]

The selective removal of the amygdala and hippocampus introduced by Neimeyer in 1958[38] was adopted by Wieser and Yasargil,[39] Olivier[40] and many others, with 80% successful arrest of seizures in selected patients.[41]

Results from the UIC on 110 patients with psychomotor epilepsy due to unilateral temporal focus (1948-1978) were reported by the senior author (JLS).[42] Operations performed under general anesthesia with electrocorticography and a 5.5-6.0 cm lobectomy sparing the amygdala and hippocampus yielded good or satisfactory results in nearly 70% patients on a 5-year follow-up.

## Temporal Lobe Epilepsy: The New Name

The introduction of the term "psychomotor epilepsy" based on the observation of a specific pattern in the EEG, which was thought to be associated with a particular variety of seizure, substantially altered nomenclature and classification debates regarding epileptic seizures involving psychic phenomena.[43] In 1951, Lennox reviewed the "psychomotor triad" (consisting of motor behaviors, automatisms with amnesia, and psychic phenomena) and advocated replacing the term with "temporal lobe epilepsy" based on clinico-anatomical observations and the results of electrical stimulation studies.[44]

## Conclusion

The development of temporal lobectomy for "psychomotor epilepsy" and the contribution of the two major teams from Montreal and Chicago can be best summarized in the words of Engel.[45]

Engel wrote:

*I was taught by Murray Falconer to always say that Bailey and Gibbs[30] were the first to perform temporal lobe resections on the basis of EEG evidence alone, but not because Gibbs was the first electroencephalographer to recognize the localizing value of anterior temporal EEG spikes, but because Bailey was the first neurosurgeon willing to operate on this evidence alone. Jasper and Kershman fully described temporal interictal EEG correlates of masticatory, olfactory, and visual auras, motor automatisms, and ictal "dreamy states" 7 years before Gibbs.[24] My own conclusion was that Jasper fully understood the importance of mesial temporal structures in the generation of what we now call complex partial seizures, but Penfield paid little attention to the EEG and directed his surgical resections entirely to removal of structural lesions*.

Finally, the routine practice of EEG recordings during sleep as an activator of temporal lobe epileptiform discharges, in addition to the initial application of stereotactic depth EEG electrodes to study epileptic patients, remains as a lasting major pivotal contribution by Gibbs to current EEG and epileptology practice.
